# An interprofessional approach to prevent recurrent obstetric anal sphincter injury (OASIS): A case report

**DOI:** 10.1002/ccr3.1834

**Published:** 2018-10-22

**Authors:** Lydia A. Thurston, Jennifer Stone, Megan Mileski, Dalia Abrams, William Huggins

**Affiliations:** ^1^ Department of Physical Therapy, College of Health Sciences Samford University Birmingham Alabama; ^2^ Department of Physical Therapy University of Missouri Healthcare Columbia Missouri; ^3^ Ida Moffett School of Nursing, College of Health Sciences Samford University Birmingham Alabama; ^4^ Birthwell Partners Community Doula Project Birmingham Alabama; ^5^ Birmingham Alabama

**Keywords:** birth, doula, dyspareunia, pelvic pain, perineum

## Abstract

Successful outcomes in this case are consistent with the American College of Obstetricians and Gynecologist (ACOG) guidelines for preventing OASIS. The interprofessional birth care team (IBCT) model exemplified by this case focuses on best practice in promoting a family's preferences for physiologic birth and preventing recurrent OASIS.

## 1 INTRODUCTION

The family in this case worked with an interprofessional birth care team skilled in applying best practice to promote the family's preference for vaginal birth and avoid recurrent OASIS. The first birth experience involved precipitous labor, obstetric anal sphincter injury (OASIS), and initiation of pelvic pain.

Data regarding the incidence of recurrent grade III‐IV perineal lesions, also termed severe perineal trauma (SPT) or obstetrical anal sphincter injuries (OASIS), are limited and sometimes conflicting.^1^, ^2^ In a 2014 Cochrane Review, the evidence assessing interventions to prevent recurrent OASIS with subsequent births was found to be inconclusive.^1^ Primary OASIS is reported to affect up to 18% (range 1.7% to 18%), and recurrent OASIS affects up to 7.2% (range 4.0% to 7.2%) of vaginal births.^1^ In the wake of inconclusive evidence, the benefits and cost‐effectiveness of doula care^3^, ^4^, ^5^, ^6^ and benefits of physiologic birth in preventing OASIS need to be revisited by students and scholars. Interprofessional birth care teams (IBCT) offer an array of skills in promoting physiologic birth. Physiologic birth is described by the American College of Nurse‐Midwives (ACNM) as including the following components: spontaneous onset and progression of labor, biological and psychological conditions that promote effective labor, vaginal birth of the infant and placenta, physiologic blood loss, and optimal newborn transition through skin‐to‐skin contact, early breastfeeding, and keeping the mother and neonate together.^7^

The purpose of presenting this case is to describe a conceptual framework for factors associated with preventing recurrent OASIS in childbirth. The model is centered around support by an IBCT that includes continuous, non‐medical support. Continuous, nonmedical support has been recommended by international health organizations, including the World Health Organization (WHO), in promoting improved birth outcomes.^8^ An explanation for resolution of pelvic pain with subsequent vaginal birth, a factor unique to this case, is also proposed. This interprofessional approach to preventing OASIS considered the health of mother and infant, and the family's preferences. Informed consent was obtained from the subject, and the case was approved by the Internal Review Board at Samford University, Birmingham, Alabama.

## 2 CASE

### 2.1 1st birth experience

The mother in this single case study design was supported through four births by an IBCT led by a family practice physician and included labor nurses and continuous nonmedical support provided by a doula and the father. The mother is Asian American, 5’ 6” with a nonpregnant BMI of 28, and college educated. Her first birth experience involved precipitous labor at 37 weeks, OASIS, and initiation of pelvic pain. Before labor began, the patient was admitted for a nonreassuring fetal heart rate pattern, with an episode at the hospital where the fetal heart rate was 60 bpm for several minutes. This fetal heart rate pattern became reassuring with oxygen and change of position by the mother from reclined to frequently changing side to side.

Nonreassuring heart rate patterns did not reoccur, and spontaneous labor began early the next day, progressing from 1 cm dilated, 50% effaced in a + 2 station to fully dilated, transition, and second stage in 25 minutes. The mother pushed during the second stage for 20 minutes. Membranes ruptured spontaneously during the second stage. During labor, the mother was unmedicated (no pain medicine or labor augmentation). Since the mother had expressed a preference prior to delivery of not birthing on her back, she was encouraged to be in a quadruped position during the pushing stage to promote labor progression. Per hospital policy, the mother pushed on command rather than with her own urge, and the mother held her breath when pushing. The newborn had an Apgar score of 9/10 at 5 minutes postdelivery, average weight at 3430.29 g (7 lbs 9 oz), and immediately initiated breastfeeding that continued for 36 months (including tandem feeding of the first and second born for 18 months). Immediate postbirth, the patient's perineum was repaired with sutures by the attending physician for what was documented at the time to be a second‐degree laceration of the perineum.

During the postpartum period, the patient experienced persistent pelvic pain that increased with sitting, voiding, and intercourse. At her 6‐week follow‐up appointment, she was told these symptoms were normal. The mother sought a second opinion at 5 months when she began menstruating and the blood was coming from the rectum. She then received a diagnosis of OASIS involving grade III pelvic floor lacerations and two rectovaginal fistulas deep in the vaginal canal. The lacerations were promptly repaired, and there was no incontinence of flatus or stool following the repair. The patient participated in physical therapy upon wound closure to address her continued complaint of severe dyspareunia and perineal/pelvic pain. Physical therapy included scar mobilization, pelvic floor muscle strengthening, and relaxation training.

After surgical repair, the patient continued to suffer from pelvic pain (reported as constant 2‐4/10), increased pain with intercourse lasting up to a day, and increased pain the week of and the week after menstruation. She reported the use of cloth hygiene pads during menstruation due to the irritation of the scar tissue with the use of other products. During this time, 10 months after the first birth and 5 months after the surgical repair, the patient became pregnant with her second child consistent with her desire for a larger family of 4‐5 children.

### 2.2 2nd birth experience

The urogynecologist who performed the OASIS repair at 5 months postpartum recommended that the mother plan to have a cesarean section (CS) at 35 weeks’ gestation to prevent recurrent OASIS with subsequent births. This plan was based on the provider's assumption that the patient would experience precipitous labor with subsequent births, an assumption that is not supported by best evidence. This family instead chose to continue to work with their interprofessional team that was skilled in and receptive to supporting the family's preference for expectant labor, continuous labor support from a doula, and perineal counter pressure during delivery. The mother performed a daily regimen of estrogen and perineal massage. In consultation with their IBCT, the family also chose to forgo epidural pain relief and allow the mother to labor in various positions, push with urge, and birth in her position of choice.

Consistent with the family's desires, the mother gave birth to a second child vaginally and without the need for surgical interventions. The mother did not receive medical pain relief during this birth. This delivery occurred at 39 weeks +2 days gestation, that is, 19 months following the first birth and 13.5 months after surgical repair and rehabilitation of the OASIS. The labor progressed over 17 hours with slow, steady dilation. In consultation with the patient, membranes were ruptured at 9 cm dilation by the family practice physician. During the second stage, the mother often labored on her side to pace the progress of the labor and she gave birth in a supine position with perineal support applied during crowning. She also pushed in short bursts with urge in the second stage of labor and avoided breath holding. Oxytocin was infused immediately after the birth of the infant and continued after the birth of the placenta. Their second child was born average weight at 3042 g (6 lbs 11 oz) with an Apgar score of 9/10 at 5 minutes and immediately initiated breastfeeding that was continued for 22 months (including tandem feeding of the first and second born for 18 months). Notably, the persistent pelvic pain and dyspareunia the mother was experiencing after the birth of her first child resolved by 10 days postpartum, with only infrequent occasions of minor pain reported. A thorough pelvic examination postdelivery determined there were no recurrent lacerations of the perineum requiring repair.

### 2.3 3rd birth experience

The family's third birth occurred 25 months following the birth of their second child. In preparation for the delivery, the mother performed the same daily regimen of estrogen and perineal massage as with the second birth. The third birth involved precipitous labor that was even more rapid than the first birth experience, laboring for 25 minutes and pushing for 5 minutes. Membranes ruptured spontaneously during the second stage, and perineal support was provided with crowning. Shoulder dystocia occurred in the second stage and resolved in 20 seconds with suprapubic pressure. Their third child was born average weight of 3884 g (8 lbs 8 oz) with an Apgar score of 9/10 at 5 minutes and immediately initiated breastfeeding that was continued for 21 months. The mother experienced minor pubic symphysis pain that resolved by 3 months postpartum with a course of physical therapy. The physical therapist interventions included myofascial release and relaxation training along with progressive strengthening as the pelvic floor muscle spasms resolved and the scar tissue became functionally mobile. No perineal lacerations were noted with a thorough postdelivery pelvic examination.

### 2.4 4th birth experience

The family's fourth birth occurred 27 months following the birth of their third child. The mother was induced at 39 weeks based on collaborative discussions between the mother and her family practice physician about avoiding a spontaneous, unassisted delivery and the suspicion of fetal macrosomia based on the 35‐week ultrasound. For this birth, a monitrice was included in the IBCT. A monitrice has a similar role as a doula in providing emotional labor support and can also provide services similar to a midwife by providing some monitoring and hands‐on support while the mother labors. Perineal massage was performed in the weeks prior to the birth without application of estrogen as the mother and physician both decided estrogen was not necessary.

Labor started 35 minutes after induction by artificial rupture of membranes. The first stage of labor was approximately 3 hours, and the second stage of labor was approximately 1.5 hours. The mother pushed in various positions, mainly a squat position, and also side‐lying and quadruped, and then she gave birth in a right side‐lying position. Perineal support with warm compress was provided with crowning. The newborn experienced shoulder dystocia that was resolved in 40 seconds with manipulation maneuvers and maternal position changes. At birth, the infant's Apgar score was 9/10 at 5 minutes, and she weighed 4646 g (10 lbs 3 oz) and immediately initiated breastfeeding that has continued to the time of manuscript submission (the infant was four months at the time of submission). The mother experienced a second‐degree laceration requiring five sutures by the family practice physician. This laceration was not along the scar from the OASIS experienced with the first birth. The mother reported no pelvic floor‐related symptoms following this birth.

## 3 DISCUSSION

**Figure 1:**
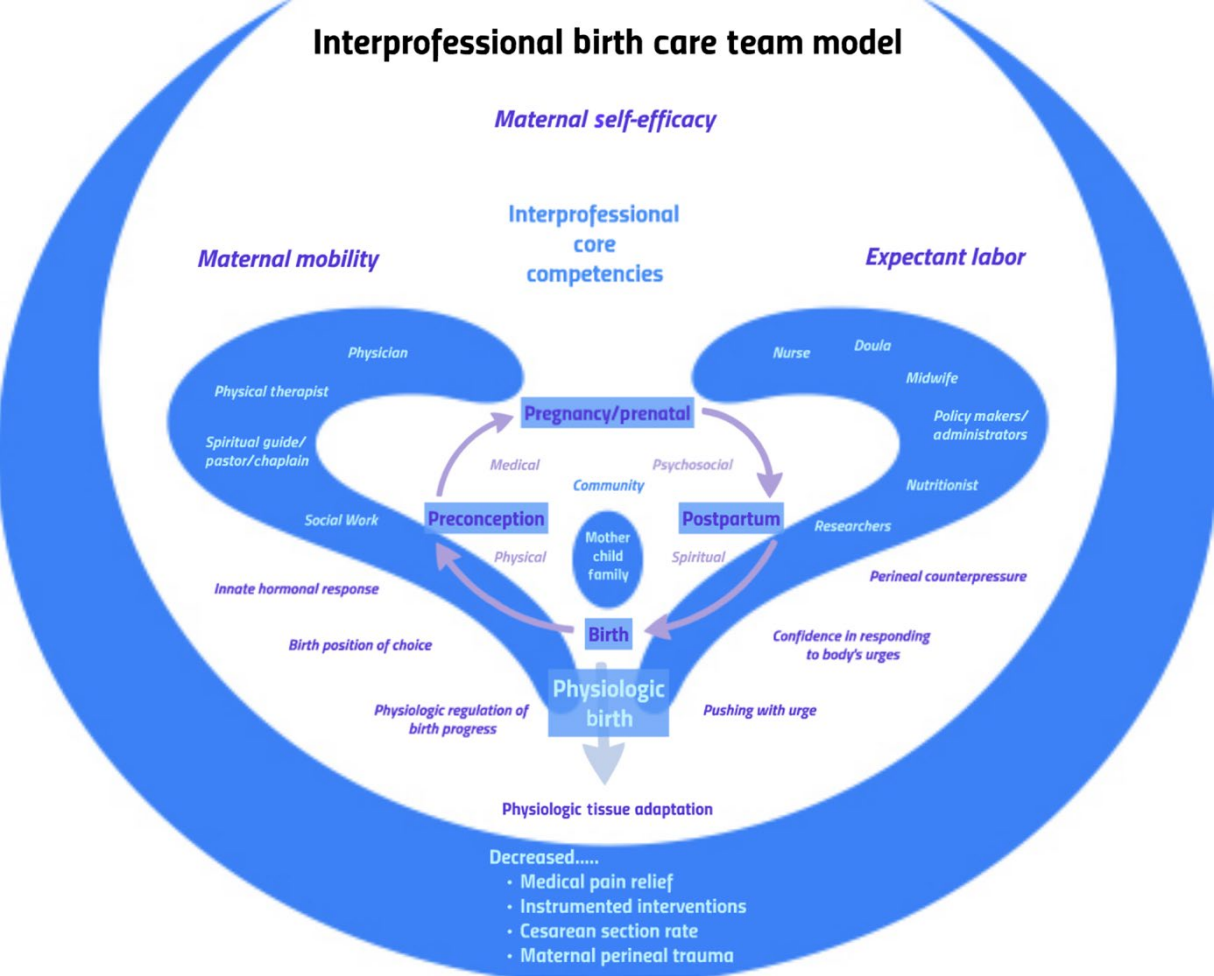
Theoretical model for best practices by an interprofessional birth care team (IBCT) in fulfilling a family's preferences for supporting physiologic birth and preventing recurrent OASIS

In light of conflicting evidence regarding primary and recurrent grade III perineal trauma,^1^ best practice is not well established for preventing OASIS. Some practitioners offer early, elective CS with subsequent births despite risks associated with CS, risks that have been reported to increase with parity.^9^, ^10^ The recommendation of CS at 35 weeks for subsequent births in this case did not involve shared interprofessional decision making and was not based on best evidence. Trauma and repair of core stabilizing structures are also requisite with CS, CS is costly, and CS is associated with a higher incidence of postpartum pain.^11^ Birth care teams that include nonmedical personnel, such as doulas, have been associated with reduced CS incidence.^4^, ^12^ In addition to reduced CS incidence and consistent with the outcomes in this case, there is evidence that birth teams with continuous labor support can optimize outcomes by improving support of physiological birth,^13^ reducing the risk of having a low birth weight (LBW) infant, reducing birth complications for the mother and infant, increasing the incidence of breastfeeding initiation,^3^ and improving cost efficiency.^14^ The authors propose a theoretical model for best practices by an interprofessional birth care team (IBCT) in fulfilling a family's preferences for supporting physiologic birth and preventing recurrent OASIS (Figure 1).

The model proposes four dimensions of care to be addressed across the cycle of childbearing from preconception to postpartum: medical, physical, spiritual, and psychosocial. The IBCT model places the mother, child, family, and their community at the center along with a variety of care providers and stakeholders.^15^ Many professionals are involved, such as healthcare policy makers that are critical to promoting policies that support evidence‐based care and collaborative practice.^16^ Working together effectively as an IBCT, three key elements are promoted: expectant labor, maternal mobility, and maternal self‐efficacy.^17^ The goal of the team in promoting these key elements is to optimize outcomes and prevent recurrent OASIS by promoting physiologic tissue adaptation to stress rather than lacerations and by decreasing the need for medical or surgical interventions.^2^, ^13^, ^18^

Consistent with the proposed model, the IBCT is bound by the Interprofessional Practice and Education Collaborative (IPEC) Core Competencies. The IPEC Core Competencies include four domains: (a) values and ethics, (b) roles and responsibilities, (c) communication, and (d) teams and teamwork.^19^ Interprofessional practice allows access to a broader array of care in supporting birth and requires training.^13^, ^20^ In addition to reducing CS incidence and consistent with the outcomes in this case, there is evidence that birth teams that include continuous labor support can optimize outcomes by improving support of physiological birth^4^ and reducing birth complications for the mother and infant.^3^, ^12^ Shared decision making among team members and in conjunction with the patient improves the self‐confidence and satisfaction of the mother^21^, ^22^ and considers the family's preferences in evaluating the evidence to determine the optimal care approach.

The three key elements, expectant labor, maternal mobility, and maternal self‐efficacy, work together to protect the perineum during birth. These key elements are supported by innate hormonal responses to labor, decreased use of medication for pain relief, and decreased use of forceps or vacuum extraction. The perineum is also protected by the use of perineal counter pressure during delivery.^2^, ^23^ In terms of the mother, these outcomes are promoted through her confidence in responding to her body's signals, thus allowing for the mother to push with urge, move into effective labor positions, and achieve physiologic regulation of birth progress.^17^ These elements complement the recommendations by the American College of Obstetricians and Gynecologists (ACOG) for prevention and management of obstetric lacerations with vaginal delivery.^2^ Evidence‐based best practice and interprofessional collaboration work together to establish effective, individualized birth care.

ACOG guidelines for management and prevention of obstetric lacerations^2^ are based on available evidence and are consistent with the currently proposed model for preventing OASIS. Measures applied in this case to prevent recurrent OASIS that were consistent with the ACOG guidelines included delaying pushing until patient urge, perineal support, warm compress, modifying labor position of the mother and restricting procedures that might lead to OASIS. Shoulder dystocia is listed as an indicator for episiotomy. The idea is that an episiotomy allows for better execution of corrective measures. The guidelines also note that differences in skill and experience levels of practitioners can impact proper execution of perineal protective measures. With this case, shoulder dystocia with the 3rd and 4th births was resolved without the need for an episiotomy.

The risk factors for OASIS listed in the ACOG guidelines include primiparity and Asian ethnicity and are consistent with the mother's race and first birth experience of OASIS. Modifiable risk factors include labor induction or augmentation, and epidural anesthesia, factors that were avoided with the first birth, yet the mother still experienced OASIS. The guidelines recommend restricting the use of operative vaginal birth techniques to only indicated cases. Operative vaginal birth techniques include vacuum extraction, use of forceps, and episiotomy. When these measures are necessary, the guidelines recommend advising the patient of the risks for perineal trauma and obtaining consent.

The ACOG guidelines also include the need for adequate lighting, exposure, and anesthesia for diagnosing deep perineal lacerations such as those experienced by the mother with her first birth.^2^ The mother in this case may not have been able to tolerate the examination necessary to identify the extent of OASIS after the first birth, leading to the initial diagnosis of only a second‐degree tear. Despite the mother reporting that she experienced excruciating pain with the immediate post‐first birth suturing, the mother insisted at the time not to have anesthetic. In contrast, after the 4th birth anesthetic was used for examination and repair of the second‐degree laceration. If the mother had a better understanding of the risks and benefits of anesthetic with birth versus with perineal repair following the first birth, she may have chosen to have pain relief with the repair. The need for physicians to learn OASIS repair skills through simulation is noted in the ACOG guidelines,^2^ and physicians need to be skilled in having conversations with patients about risks and benefits of pain relief. Although not highlighted in the guidelines, the use of authentic scenarios, such as this case, may be beneficial in simulation for practicing advanced delivery procedures and communication skills.

The success of interprofessional birth care initiatives are well established in the literature.^15^, ^24^, ^25^, ^26^, ^27^ The ACOG‐ACNM Project identified essential components of successful collaborative maternity care models through analysis of 12 articles. A culture of mutual respect and trust were identified as key components of sustainable collaborative care models, and this was best facilitated through institutions emphasizing effective team work. Regulation that allows care providers to practice the full scope of their training was also noted as being ideal for successful collaboration. The ACOG‐ACNM Project established that cohesive healthcare teams are associated with improved clinical outcomes and higher patient satisfaction,^15^ as demonstrated by this case.

Grumbach & Bodenheimer pointed out a number of barriers to team formation, including relationship and personality conflicts.^28^ The high stakes associated with birth may be an added barrier to effective team dynamics and shared decision making and certainly contributed to the course of the first birth experience in this case. The first birth experience was at times emergent, and protection of life had to be prioritized. Healthcare policies can either be a facilitator or barrier to shared decision making.^16^ The providers in the first birth experience considered the mother's preferences when possible and successfully avoided CS. However, at times, factors were dictated by hospital policy rather than decided by shared decision making. For example, pushing on command was a hospital policy that promoted breath holding with the first birth. It is conceivable that the outcome of perineal trauma may have been primarily related to the patient's risk factors, including primiparity and Asian race.^18^ The mother labored in her desired position of quadruped, though this position may not have been ideal in protecting the perineum. The supine position or a sidelying position, as with subsequent births, may have slowed labor progression and been protective. The patient's desire for no pain relief was respected by the team, though perineal repair without adequate anesthetic may have made it difficult to assess the true extent of perineal trauma. Notably, by avoiding a CS during her first birth, the mother was able to realize her preferences for subsequent vaginal births. Those subsequent vaginal births were managed by an IBCT engaged in shared decision making based on best evidence.

The mother in this case reported a resolution of her pelvic pain with her second vaginal birth. This is an important factor to note in this case since it is rational to conclude that vaginal birth might worsen pelvic pain. This case demonstrates that a vaginal birth does not necessarily aggravate pelvic pain conditions and could be a factor in resolving pelvic pain in some instances. Although the reason for pain resolution is not clear with this case, spontaneous release of scar tissue with vaginal birth seems to be the most likely explanation. The use of estrogen and perineal massage prior to subsequent deliveries could be another explanation for both the prevention of OASIS and pain resolution, though these measures are not supported by evidence and estrogen levels are already high prior to delivery.^29^ The release may have been facilitated by the slow, steady course of the birth and allowing the mother to push with urge rather than on command.

The resources for an IBCT were available to this family where the mother had a post‐secondary education, supportive family, and employer‐sponsored private health insurance. These factors are associated with favorable birth outcomes and are consistent with reports of disparities associated with race and socioeconomic status.^30^ The parents in this case had a high level of health literacy and were engaged in their healthcare decision‐making process. In contrast, for parents with lower levels of health literacy and limited access to healthcare services, adverse birth outcomes are associated with decreased engagement in prenatal care.^31^ Birth teams that include doula care have been shown to improve birth outcomes among low‐resource women, a population with recognized birth health disparities.^32^, ^33^ Currently, the models for providing doulas to low‐resource women mainly involve nonprofit organizations.^34^ Families with limited resources are at most risk for adverse birth outcomes and are the least likely to have access to a comprehensive birth team. Sustainable funding for IBCTs is of particular interest for families with limited resources.

## 4 CONCLUSION

The favorable outcomes in this case and the limited evidence on best practices for preventing recurrent OASIS highlight the need to overcome barriers to comprehensive and cohesive interprofessional birth teams, even in emergent situations. Consistent implementation of best practices to prevent recurrent OASIS requires open dialogue between the family and an IBCT skilled in integrating those preferences. Evidence is needed to support funding and wide use of IBCTs that can include continuous labor support from nonmedical providers (doulas), regardless of family resources. Educational institutions have the challenge of developing birth simulations that are realistic enough to translate successful components of collaboration into practice, and include all the elements of a mobile patient during birth. Interprofessional research, practice, and education collaborations are tools for mitigating recurrent OASIS with childbirth and facilitating family‐centered care. This is an authentic case that can be used in healthcare education to promote interprofessional birth care practices, shape student perspectives related to the complexity of managing OASIS, and as a basis for research endeavors.

## CONFLICT OF INTEREST

None declared.

## **AUTHOR CONTRIBUTIO**N

All authors were involved in drafting or revising the content of this manuscript, providing intellectual content and critical analyses, and providing final approval. LT: the main author who provided primary development and revision of the manuscript. LT and JS: both physical therapists with specialty training in pelvic health, had full access to the original case data and were responsible for the initial concept. MM: provided input relative to expertise in perinatal nursing. DA: provided the perspective of a doula and doula trainer with a Master of Public Health degree. WH: the senior author, provided his perspective as an obstetrician and gynecologist.

## References

[1] Farrar D , Tuffnell DJ , Ramage C . Interventions for women in subsequent pregnancies following obstetric anal sphincter injury to reduce the risk of recurrent injury and associated harms. Cochrane Database Syst Rev. 2014(11):CD010374. 25373366 10.1002/14651858.CD010374.pub2 PMC10823349

[2] American College of Obstetricians and Gynecologists' Committee on Practice, Bulletins‐Obstetrics . Practice bulletin no. 165: prevention and management of obstetric lacerations at vaginal delivery. Obstet Gynecol. 2016;128(1):e1–e15. 27333357 10.1097/AOG.0000000000001523

[3] Kozhimannil KB , Hardeman RR , Attanasio LB , Blauer‐Peterson C , O'Brien M . Doula care, birth outcomes, and costs among Medicaid beneficiaries. Am J Public Health. 2013;103(4):e113–e121. 23409910 10.2105/AJPH.2012.301201 PMC3617571

[4] Gruber K , Dobson C . Impact of healthy birth outcomes. J Perinat Educ. 2013;22(1):49‐58. 24381478 10.1891/1058-1243.22.1.49 PMC3647727

[5] Hodnett ED , Gates S , Hofmeyr GJ , Sakala C . Continuous support for women during childbirth. Cochrane Database Syst Rev. 2013;7:CD003766. 23857334 10.1002/14651858.CD003766.pub5

[6] Nommsen‐Rivers LA , Mastergeorge AM , Hansen RL , Cullum AS , Dewey KG . Doula care, early breastfeeding outcomes, and breastfeeding status at 6 weeks postpartum among low‐income primiparae. J Obstet Gynecol Neonatal Nurs. 2009;38(2):157‐173. 10.1111/j.1552-6909.2009.01005.x 19323712

[7] Nurse‐Midwives ACo . Birthtools: What is physiologic birth? http://birthtools.org/What-Is-Physiologic-Birth. Accessed June 5, 2018.

[8] Duff E . WHO Safe Childbirth Checklist. Midwifery. 2016;33:8. 27294231

[9] Marshall NE , Fu R , Guise JM . Impact of multiple cesarean deliveries on maternal morbidity: a systematic review. Am J Obstet Gynecol. 2011;205(3);262. e261–268. 10.1016/j.ajog.2011.06.035 22071057

[10] Edozien LC , Gurol‐Urganci I , Cromwell DA , et al. Impact of third‐ and fourth‐degree perineal tears at first birth on subsequent pregnancy outcomes: a cohort study. BJOG. 2014;121(13):1695‐1703. 25040835 10.1111/1471-0528.12886

[11] Kainu JP , Halmesmaki E , Korttila KT , Sarvela PJ . Persistent pain after cesarean delivery and vaginal delivery: a prospective cohort study. Anesth Analg. 2016;123(6):1535‐1545. 27870738 10.1213/ANE.0000000000001619

[12] Kozhimannil KB , Attanasio LB , Jou J , Joarnt LK , Johnson PJ , Gjerdingen DK . Potential benefits of increased access to doula support during childbirth. Am J Manag Care. 2014;20(8):e340–e352. 25295797 PMC5538578

[13] Zielinski RE , Brody MG , Low LK . The value of the maternity care team in the promotion of physiologic birth. J Obstet Gynecol Neonatal Nurs. 2016;45(2):276‐284. 10.1016/j.jogn.2015.12.009 26820357

[14] Strauss N , Giessler K , McAllister E . How doula care can advance the goals of the affordable care act: a snapshot from New York city. J Perinat Educ. 2015;24(1):8‐15. 26937157 10.1891/1058-1243.24.1.8 PMC4720857

[15] Avery MD , Montgomery O , Brandl‐Salutz E . Essential components of successful collaborative maternity care models: the ACOG‐ACNM project. Obstet Gynecol Clin North Am. 2012;39(3):423‐434. 22963701 10.1016/j.ogc.2012.05.010

[16] Shaw D , Guise JM , Shah N , et al. Drivers of maternity care in high‐income countries: can health systems support woman‐centred care? Lancet. 2016;388(10057):2282‐2295. 27642026 10.1016/S0140-6736(16)31527-6

[17] Saftner MA , Neerland C , Avery MD . Enhancing women's confidence for physiologic birth: Maternity care providers' perspectives. Midwifery. 2017;53:28‐34. 28743051 10.1016/j.midw.2017.07.012

[18] Pergialiotis V , Vlachos D , Protopapas A , Pappa K , Vlachos G . Risk factors for severe perineal lacerations during childbirth. Int J Gynaecol Obstet. 2014;125(1):6‐14. 24529800 10.1016/j.ijgo.2013.09.034

[19] Schmitt M , Blue A , Aschenbrener CA , Viggiano TR . Core competencies for interprofessional collaborative practice: reforming health care by transforming health professionals' education. Acad Med. 2011;86(11):1351. 22030650 10.1097/ACM.0b013e3182308e39

[20] Priddis HS , Schmied V , Kettle C , Sneddon A , Dahlen HG . "A patchwork of services"–caring for women who sustain severe perineal trauma in New South Wales–from the perspective of women and midwives. BMC Pregnancy Childbirth. 2014;14:236. 25034120 10.1186/1471-2393-14-236 PMC4223519

[21] Meadow SL . Defining the doula's role: fostering relational autonomy. Health Expect. 2015;18(6):3057‐3068. 25327532 10.1111/hex.12290 PMC5810740

[22] Moore JE . Women's voices in maternity care: the triad of shared decision making, informed consent, and evidence‐based practices. J Perinat Neonatal Nurs. 2016;30(3):218‐223. 27465453 10.1097/JPN.0000000000000182

[23] Schmittdiel J , McMenamin SB , Halpin HA , et al. The use of patient and physician reminders for preventive services: results from a National Study of Physician Organizations. Prev Med. 2004;39(5):1000‐1006. 15475035 10.1016/j.ypmed.2004.04.005

[24] Brady K , Bulpitt D , Chiarelli C . An interprofessional quality improvement project to implement maternal/infant skin‐to‐skin contact during cesarean delivery. J Obstet Gynecol Neonatal Nurs. 2014;43(4):488‐496. 10.1111/1552-6909.12469 PMC4491370 24981767

[25] Kapoor DS , Sultan AH , Thakar R , Abulafi MA , Swift RI , Ness W . Management of complex pelvic floor disorders in a multidisciplinary pelvic floor clinic. Colorectal Dis. 2008;10(2):118‐123. 18199292 10.1111/j.1463-1318.2007.01208.x

[26] Mottl‐Santiago J , Fox CS , Pecci CC , Iverson R . Multidisciplinary collaborative development of a plain‐language prenatal education book. J Midwifery Womens Health. 2013;58(3):271‐277. 23647968 10.1111/jmwh.12059

[27] Vedam S , Leeman L , Cheyney M , et al. Transfer from planned home birth to hospital: improving interprofessional collaboration. J Midwifery Womens Health. 2014;59(6):624‐634. 25533708 10.1111/jmwh.12251

[28] Grumbach K , Bodenheimer T . Can health care teams improve primary care practice? JAMA. 2004;291(10):1246‐1251. 15010447 10.1001/jama.291.10.1246

[29] Tsesis S , Gruenbaum BF , Ohayon S , et al. The effects of estrogen and progesterone on blood glutamate levels during normal pregnancy in women. Gynecol Endocrinol. 2013;29(10):912‐916. 23862584 10.3109/09513590.2013.813467

[30] Lu MC , Halfon N . Racial and ethnic disparities in birth outcomes: a life‐course perspective. Matern Child Health J. 2003;7(1):13‐30. 12710797 10.1023/a:1022537516969

[31] Bennett I , Switzer J , Aguirre A , Evans K , Barg F . 'Breaking it down': patient‐clinician communication and prenatal care among African American women of low and higher literacy. Ann Fam Med. 2006;4(4):334‐340. 16868237 10.1370/afm.548 PMC1522153

[32] Kozhimannil KB , Attanasio LB , Hardeman RR , O'Brien M . Doula care supports near‐universal breastfeeding initiation among diverse, low‐income women. J Midwifery Womens Health. 2013;58(4):378‐382. 23837663 10.1111/jmwh.12065 PMC3742682

[33] Steel A , Frawley J , Adams J , Diezel H . Trained or professional doulas in the support and care of pregnant and birthing women: a critical integrative review. Health Soc Care Community. 2015;23(3):225‐241. 24942339 10.1111/hsc.12112

[34] Thomas M , Ammann G , Brazier E , Noyes P , Maybank A . Doula services within a healthy start program: increasing access for an underserved population. Matern Child Health J. 2017;21(Suppl 1):59‐64. 29198051 10.1007/s10995-017-2402-0 PMC5736765

